# Supplementary material for: Social protection and informality in Latin America during the COVID-19 pandemic
Source: PLoS One. 2021 Nov 4;16(11):e0259050. doi: 10.1371/journal.pone.0259050 (PMC8568185; doi:10.1371/journal.pone.0259050)
Supplement: S1 File — (ZIP) [file pone.0259050.s001.zip › SupportingInfoS1File_TrackChanges.pdf]

Supporting Information

Identifying COVID-19 emergency measures and lockdown periods

We used several websites and news sources to identify the policies put in place in each country. Policies were identified primarily through official government websites that track countries' COVID-19 policy responses. S1 Table offers a full list of these websites. Sometimes the official information is incomplete or updated with a delay. All the information was cross-checked with several sources. The main newspapers in each country were scraped, searching for the following keywords: "subsidy", "transfer", "coronavirus", "bonus", and "aid". We also checked policy makers' weekly response against the COVID-19 database put together by the COVID-19 Policy Measures Team at the Inter-American Development Bank (IDB). The last source for double checking and obtaining updates was the ACAPS COVID-19: Government Measures Dataset. All the sources are included in S1 Table.

Our analysis focuses exclusively on policies implemented during the lockdown period in each country. Table 2 in the main text presents a detailed overview of the policies implemented by the 10 countries. We gathered information related to the beneficiaries (households or individuals), the amount and frequency of the emergency cash transfers, and the eligibility criteria. There is great heterogeneity in the measures taken by governments concerning the implementation date, frequency of payments (unique lump sum or monthly) and lockdown duration.

We defined as lockdown the period when stay-at-home measures were implemented or strongly suggested (in the case of Uruguay), and non-essential sectors were shut down. Establishing the start of the quarantine is straightforward. However, determining the end-date of the lockdown is more difficult. We use the date when countries allowed some non-essential sectors to start operating. Because Brazil and Chile never implemented a national lockdown, we consider as start- and end-dates the lockdowns of their main economic region: the State of Sao Paulo in Brazil, and the Region Metropolitana de Santiago in Chile.

Matching emergency programs to household surveys and calculating the Replacement Rate

We identified the beneficiaries of each program using the individual and household characteristics described in the program and the equivalent information from the 2018 household surveys harmonized by the IDB (except in Chile, where the household survey is bi-annual and the latest available wave was 2017). See S2 Table for a full list of the surveys used. Table 2 shows that the beneficiaries of 19 programs could be directly mapped with household survey information. This is the case for most programs that expand pre-existing social programs whose beneficiaries can be identified in the survey (such as Juntos or Pension 65 in Peru) or target specific segments of the population that have a one to one correspondence with information in the survey (like Bono Familia in Bolivia, that targets families with children attending primary and secondary education). In 12 programs the correspondence was not direct, which obliged us to approximate the beneficiaries. If the program excludes a certain segment of the population that cannot be identified in the survey, we simply ignore the restriction. This makes our estimates of

potential coverage and replacement rate effectively an upper bound. The assumptions are listed as follows:

- *Argentina.* We approximated Policies (2), (4) and (6), since the survey does not have a variable to identify them directly. Policies (2) and (4) aim at Asignacion Universal por Hijo (AUH) beneficiaries, which is a program for unemployed; informal economy workers with incomes less than a minimum wage; a special category of taxpayers (monotributistas sociales); domestic service workers; and beneficiaries of Hacemos Futuro, Manos a la Obra, and Ministry of Labor (Secretaria de Gobierno de Trabajo) programs. All beneficiaries must have at least one child younger than 18 years. We were able to map most of all the conditions, except, due to lack of taxpayer data, we could not identify the specific taxpayers (monotributistas), instead we proxied it by including all the self-employed that fulfill the rest of the conditions.
  Policy (6), Ingreso Familiar de Emergencia, targets informal, domestic service and *monotributista social* in categories A-B household heads without unemployment aid or who have a dependent source of income in the household. As we could not identify the taxpayer criteria, we proxied it by including self-employed household heads earning less than ARS$26,092 per month. This aid is automatically given for AUH and PROGRESAR beneficiaries. As we could not identify the latter beneficiaries using the survey, we proxied them by assuming that all individuals who are elegible for PROGRESAR are beneficiaries. Hence, we include any household with at least one member between ages 18 and 24, who is not employed and with a household monthly total income less than three times the minimum wage, and who are enrolled in an educational program.

- *Brazil.* We made assumptions for policies (10) and (11). Policy (10) was aimed to help self-employed and informal workers who cannot work during the social distancing restrictions, especially informal households who are not part of Bolsa Familia. We assumed that all households that have at least one of the target beneficiaries receive R$1,200. We approximate this population by households with a single female household head and at least one child, and households whose household head works as an informal worker, is self-employed, or is an employer in a firm with 0-5 workers (microentrepreneurs). All households that are beneficiaries of this program must have total incomes less than R$3,135.
  Policy (11) targets Bolsa Familia Beneficiaries. The government gave them the option to receive R$1,200 if it was higher than their usual transfer. Therefore, we assumed that both Bolsa Familia beneficiaries and informal households receive R$1,200. We could not identify Bolsa Familia beneficiaries directly from the survey. Therefore, we approximated it with households under extreme poverty and households below the 2018 poverty line who have at least one child between 0 and 17 years.

- *Colombia.* Policy (15), Ingreso Solidario, targets households in extreme poverty and poverty identified in The System of Identification of Social Programs (SISBEN) that do not receive any social program. The survey data does not contain an indicator variable for households under the poverty line in the SISBEN, but SISBEN uses national poverty lines. We identify the beneficiaries as those households below the 2018 national poverty and extreme poverty lines.

- *Chile.* The design of Policies (16 - 18) uses country-specific indexes or indicators, such as Registro Social de Hogares (RSH) and the Indicador Socioeconomico de Emergencia (ISE) to target vulnerable households. The household survey does not have these indicators. Scores in the RSH are approximated by household income

per capita. Because we cannot approximate the ISE indicator, we extend the coverage to all households who meet the rest of the criteria.
Policy (16) originally established three subgroups of beneficiaries receiving differential monthly aid according to their vulnerability level and the number of household members. The first two subgroups were a three-month aid program, which targets 90% of the most vulnerable households. We approximated it by including households below the 90th percentile of total income per capita. They also used as criteria the 60% and 40% most vulnerable according to the ISE. As we could not observe it in the data or approximate it, we excluded this condition. The first subgroup received 100% of the cash aid, as it is also aimed at households without formal workers. The second subgroup received 50% of the benefit, as it is aimed at households with mainly informal income and income less than the aid they would receive if they were in the first subgroup. To avoid using an arbitrary criterion of main income, in the second subgroup, we considered households with at least one formal and one informal worker. Lastly, the third subgroup targets for the 80% most vulnerable households with at least one older adult benefiting from the Pension Basica Solidaria Vejez. The survey allowed us to identify the last two conditions but not the first, which we approximated using total income per capita. Furthermore, originally for all three subgroups, the transfer amount decreased each month, meaning that the first monthly transfer will be the highest of the whole. However, after the first transfer and during the lockdown period, Chile redesigned this policy to increase its coverage and aid. Now, the first group includes those households registered in the RSH without any formal household members. These households receive 100 000 CLP per capita until it reaches 10 household members (In this case it receives 759 000 CLP). The second group also needs to be registered in the RSH and includes those households with at least 1 formal worker. The amount given depends on the total formal income the household receives and the number of household members. The third group now includes more older adults, those who benefit from Basic Solidarity Pension for Old Age or invalidity or beneficiary of Solidarity Social Security Contribution for Old Age or Disability. With this new design, the policy aims to provide a higher payment during the second, third, and four months. After, it will decrease for the fifth and sixth months. For this analysis, during the lockdown period, only three payments were implemented.

Policy (17), Bono Invierno, was a unique transfer allocated to retired people. The variables in the survey data allow us to identify some of the beneficiaries directly, but there are some who are excluded for lack of data.

For policy (18), the Covid- 19 emergency bonus, we used the survey to directly identify the beneficiaries of Asignacion Familiar program (SUF) and those who are part of the SSyOO database. Nonetheless, we approximated the 60% most vulnerable households according to the RSH database by including all households earning below the 60th percentile of the total income per capita.

- *El Salvador*. Policy (19) is designed to aid informal households and to cover at least 70% of the workers. Hence, we approximated informality as individuals without pension benefits or who were not affiliated to social security and, to reach the 70% objective, we also included in our proxy formal self-employed workers. Initially, the policy was designed to provide a monthly payment. However, up until October, only one payment had been transferred. Therefore, we consider it as a one-time payment.

- *Ecuador*. Policy (20) has several subgroups of beneficiaries such as Seguro Social Campesino beneficiaries and affiliated to the unpaid work regime. We were able to

identify the first group but not the latter. We approximated unpaid workers by including all people working as nonremunerated and assuming everyone is under this regime. We also included self-employed workers as it has been stated that this is one of the policy objectives. Accordingly, following the policy design, we restricted the beneficiaries to have a labor income less than US$400, and not receive other government transfers, such as Bono de Desarrollo Humano or disability transfers, and exclude those who are not contributing to social security. We restricted the sample of beneficiaries to be 18 or older.

- *Dominican Republic.* Policy (27) was a transfer intended for vulnerable and poor households according to SIUBEN (country-specific targeting indicator). We approximated the potential beneficiaries using the 2018 national poverty line.
- *Uruguay.* For Policies (29) and (31) the survey had specific variables for directly identifying the beneficiaries. In Policy (29), we identify Tarjeta Uruguay Social beneficiaries and assign the current 2020 amount according to the number of children in the household. Nonetheless, as we could not identify whether there is a pregnant woman in the household, we only assigned the extra transfer of UYU$292 per child in the household and exclude the pregnant women benefits. Policy (31), Plan Equidad, is a conditional cash transfer program. The amount given depends on the number of eligible beneficiaries in the household and educational level, among others. The household survey allowed us to identify the targeted households but not the amount received. To avoid trying to approximate the amount each household receives, as it depends on a list of variables we often do not observe (for example, if the first beneficiary in the household was pregnant or was a child under five years old), we relied on the amount already stated in the survey but we inflated it to 2020 prices, using the inflation rates of 2019 and up to April 2020.

Once beneficiaries were identified, we simulated the total household transfer for each household by adding all the individual and household transfers received by each eligible member.

Calculating the replacement rate of each program required some manipulation. All transfers were transformed into the monthly equivalent for the lockdown period. When the transfer was a lump sum we used the monetary amount of each program and duration of the lockdown to calculate the equivalent monthly transfer. When the transfer was monthly for an initial lockdown period that was later expanded (without expanding the program), we pro-rated the monetary transfer for the duration of the lockdown period. In those countries where the transfer was monthly and was in place during the entire lockdown period -such as Brazil, Colombia, and Uruguay- no transformation was needed.

The replacement rate is calculated as the ratio of the monthly emergency transfer with respect to the normal labor income obtained by the household in 2018 (2017 in Chile). Hence, we restricted the analysis to a sample of households with a monetary labor income per capita greater than zero, equivalent to 80 percent of the households. Because transfers are in 2020 prices, we transformed usual labor earnings to 2020 prices using the official inflation rates.

S1 Table. Covid-19 policies sources

**Sources**
**1. Government web pages**
https://coronavirusrd.gob.do/ (Dominican Republic)<br>https://www.argentina.gob.ar/coronavirus/medidas-gobierno (Argentina)<br>https://www.argentina.gob.ar/economia/medidas-economicas-COVID19/ingresofamiliardeemergencia (Argentina)<br>https://www.argentina.gob.ar/noticias/el-gobierno-oficializo-la-segunda-etapa-del-pago-del-ife (Argentina)<br>https://www.boliviasegura.gob.bo/normativa.php (Bolivia)<br>https://id.presidencia.gov.co/especiales/200317-medidas-enfrentar-coronavirus/index.html (Colombia)<br>https://coronaviruscolombia.gov.co/Covid19/acciones/acciones-de-economia.html (Colombia)<br>https://www.chileatiende.gob.cl/fichas/77255-bono-de-emergencia-covid-19 (Chile)<br>https://www.gob.pe/busquedas?contenido[]=noticias&institucion[]=presidencia&reason=sheet&sheet=1 (Peru)<br>https://www.gub.uy/ministerio-salud-publica/coronavirus (Uruguay)<br>https://www.economiayfinanzas.gob.bo/ (Bolivia)<br>https://calendariobolsafamilia2020.net/quem-tem-direito-ao-bolsa-familia-2020/amp/ (Brazil)<br>https://www.presidencia.gob.sv/presidente-nayib-bukele-confirma-la-entrega-del-subsidio-de-300-a-750000-familias/ (El Salvador)<br>https://www.produccion.gob.ec/wp-content/uploads/2020/03/Decreto_Ejecutivo_no._1022_20200227194449_compressed1.pdf (Ecuador)<br>https://www.gub.uy/ministerio-desarrollo-social/comunicacion/comunicados/beneficios-otorgados-monotributistas-sociales-mides (Uruguay)<br>https://www.ingresodeemergencia.cl/faq (Chile)<br>https://www.chileatiende.gob.cl/fichas/78385-ingreso-familiar-de-emergencia (Chile)<br>https://ingresosolidario.dnp.gov.co/ (Colombia)<br>https://www.inclusion.gob.ec/gobierno-nacional-entregara-bono-de-contingencia-a-400-mil-familias-por-la-emergencia-sanitaria/<br>https://minpre.gob.do/comunicacion/notas-de-prensa/gobierno-anuncia-entrada-a-fase-2-desescalada-a-partir-de-manana-tasa-de-contagio-se-ha-aplanado-y-letalidad-es-una-de-las-mas-bajas/<br>https://coronavirusrd.gob.do/2020/04/26/listado-de-medidas-rd-vs-covid-19/<br>https://www.presidencia.gub.uy/comunicacion/comunicacionnoticias/lacalle-pou-conferencia-nueva-normalidad
**2. Newspapers**
El Comercio, El Tiempo, El Universo, El Peruano, El Observador, El Deber, La Nacion, La Republica, ElSalvador.com, El Desconcierto, Diario Financiero, Infobae, El Clarin, Valor Investe - Globo Noticias, Agencia Brasil, Reuters, Correio Braziliense,Prensa Libre, Ciento Ochenta (180), La Diaria, Mega Noticias - Chile, Bolivia Emprende, Periodico Bolivia, Libero, Opinion - Bolivia, El Economista, Diario El Mundo, Gestion - Peru, Republica - Uruguay, France24, TVPeru, Listin diario.
**3. General datasets and publications**
ACAPS COVID-19 Dataset (URL: https://data.humdata.org/dataset/acaps-covid19-government-measures-dataset)<br>Americas Society Council of the Americas Where is the Coronavirus in Latin America? from May 21, 2020 (https://www.as-coa.org/articles/where-coronavirus-latin-america)<br>IADB COVID-19 policy Measures by the Covid-19 policy Measures Team<br>https://www.ey.com/es_cr/tax/medidas-especiales-covid-19
**4. Inflation data**
https://rpp.pe/economia/economia/inei-peru-registro-una-inflacion-de-19-en-2019-noticia-1237290 (Peru)<br>Instituto Nacional de Estadistica Informatica (INEI - Peru)<br>Banco Central de Bolivia (Bolivia)<br>Banco de la Republica (Colombia)<br>Instituto Nacional de Estadistica (Bolivia)<br>Instituto Nacional de Estadistica (Uruguay)<br>Instituto Nacional de Estadistica y Censos (Ecuador)<br>Instituto Nacional de Estadistica y Censos (Argentina)<br>Instituto Brasileiro de Geografia e Estatistica (Brazil)<br>Banco Central (Dominican Republic)<br>Banco Central de Reserva (El Salvador)<br>Instituto Nacional de Estadistica (Chile)

S2 Table. Household Surveys and Year by Country studied

Country	Household survey	Year
Argentina	Encuesta Permanente de Hogares (EPH)	2018
Bolivia	Encuesta Continua de Hogares (ECH)	2018
Brazil	Pesquisa Nacional por Amostra de Domiciolios (PNAD)	2018
Chile	Encuesta de Caracterizacion Socioeconomica Nacional (CASEN)	2017
Colombia	Gran Encuesta Integrada de Hogares (GEIH)	2018
Dominican Republic	Encuesta Nacional de Hogares - Fuerza de Trabajo (ENHFT)	2018
Ecuador	Encuesta Nacional de Empleo, Desempleo y Subempleo (ENEMDU)	2018
Peru	Encuesta Nacional de Hogares sobre Condiciones de Vida y Pobreza (ENAHO)	2018
El Salvador	Encuesta de Hogares de Propositos Multiples (EHPM)	2018
Uruguay	Encuesta Continua de Hogares (ECH)	2018
