# Supplementary material for: Social protection and informality in Latin America during the COVID-19 pandemic
Source: PLoS One. 2021 Nov 4;16(11):e0259050. doi: 10.1371/journal.pone.0259050 (PMC8568185; doi:10.1371/journal.pone.0259050)
Supplement: Attachment — Submitted filename: Response to Reviewers.pdf [file pone.0259050.s003.pdf]

Response to reviewers [PONE-D-21-12981]

We thank the editor and both reviewers for their constructive comments and insights which we consider have allowed us to improve the paper and make it more relevant to PLOS One's readers. Below we detail our responses to each of the comments and suggestions.

Responses to comments by the Editor

C1: *Both reviewers reported the piece to be strong and a good addition to the literature. You will note that reviewer 1 felt that the piece may be better suited to another journal. Following clarification with the PLOS One editorial office, we feel that the piece meets the publication criteria of PLOS One but I would like you to add (perhaps as a paragraph/section) a brief critical appraisal of the evidence relating to the health and public health impacts of the cash transfer schemes, including on health inequalities if possible. We think that this would strengthen the manuscript and make it even more relevant for PLOS One readership. It would also be beneficial to researchers and policy makers in the field of medicine, especially poverty-related diseases such as tuberculosis, to have a summary of this evidence with which to guide future strategies.*

R1: Thank you for the comment which has allowed us to further expand the link of our research to the public health literature. We have added two paragraphs in the introduction. The new paragraph #2 discusses the previous literature on the potential effects of income losses on health outcomes. We also added a new paragraph #4 which presents existing evidence on the effect of unconditional cash transfers, like the ones analyzed in our study, on health outcomes both of children and adults.

Responses to comments by Reviewer #2

C1: *"I think that in order to meet better the requirements of this journal there should be a more explicit health focus or at least a focus on the public health impact of the interventions you discuss."*

R1: Thank you for the comment which has allowed us to further expand the link of our research to the public health literature. We have added two paragraphs in the introduction. The new paragraph #2 discusses the previous literature on the potential effects of income losses on health outcomes. We have also added a new paragraph #4 which presents existing evidence on the effect of unconditional cash transfers, like the ones analyzed in our study, on health outcomes both of children and adults.

C2:"*I wonder whether your analysis could be complemented to meet either of the above, for example by attempting to see whether the coverage and income replacement achieved correlate with the severity of the pandemic (in terms of morbidity and mortality) or even the duration of it. In other words, in my opinion what is missing in the paper is a more obvious link with either the containment of the pandemic or the mitigation of the health damaging effect of the control measures. For example, authors could try to link the data they present (even only ecologically) with the exacerbation of health inequalities or some potential excess in the burden of other diseases (resulting from the social distancing measures) in these countries. A research question could be: do country with a better income replacement rate show also better covid morbidity and mortality or more stable health indicators (i.e. suicide, alcohol use, violence and other indicators that are sensitive to financial shocks). In conclusion, this is an extremely interesting and potentially useful paper. I just don't think Plos is a good fit for it or it could become a good fit by adding a third piece of analysis able to look at how the findings of this paper (in terms of coverage and income replacement) correlate with a better or worse success in the management of the pandemic.The above could be done for all countries ideally, or pick up one or two countries to be more in depth case studies.*"

R2: We agree that this is an excellent research question. However, we believe it is beyond the scope of the paper. We felt that adding this section may render the paper less focused. Perhaps more importantly, with the data at hand it would be very difficult to address this question appropriately. To the extent of our knowledge, there is no data on the income level of people that were infected or died of COVID-19. This implies that we could only exploit country-level variation. Because we only have 10 countries in our analysis, our ability to control for (observable) omitted variables, even in an ecological study, would be very limited. Note also that our analysis does not report the actual coverage

or replacement rates of the social protection programs, while case counts, daily deaths and other health indicators (e.g. suicides) refer to actual events. We believe a proper analysis of the linkages between social protection programs and health indicators during the COVID-19 pandemic would be much more interesting once household surveys for 2020 become available and the real incidence of social protection programs is observed. Perhaps this analysis could exploit within-country variation to control for observable (and unobservable) confounding factors.

Responses to comments by Reviewer #3

C1:*The following sentence should be changed to reflect that informal sector work excludes populations from "contributory" government safety nets. Indeed, many large social safety nets in LAC which are non-contributory (Bolsa Familia, PROSPERA, etc. do include informal sector workers. "These households to a large extent work in the informal sector, which excludes them from government safety nets."*

R1: Thank you for the comment. This is an important distinction and we have therefore included the word "contributory" in that sentence to make it more precise.

C2."*Authors could add language on vertical expansion (to existing participants) and horizontal expansion (to those who might fall poor) in their discussion of potential coverage on page 2: "potential to be expanded in times of crisis to make transfers more generous for those who are structurally poor. At the same time, it demonstrates the limitations of those safety nets to reach those who might fall into poverty temporarily"*

R2: Thank you. This is a very nice idea. We have edited the last paragraph of page 3. The relevant sentence now reads: "Our paper assesses the suitability of these programs to respond to an unexpected crisis. It shows that the existing safety nets have the potential to be vertically expanded in times of crisis to make transfers more generous for those who are structurally poor and, thus, most likely to already be beneficiaries of those programs. At the same time, it demonstrates the limitations of those safety nets to be expanded horizontally to reach those who might fall into poverty temporarily."

C3. "*I suggest changing the language of "significant fraction" – it makes it sound large, but I think the authors are saying the fraction is still generally small.*"

R3. Thank you for pointing out this mistake which has now being fixed.

C4. "*Section 1. On page 4, where authors say "no household member contributes to social security," social security can mean different things in different countries. Would "contributory social protection programs" be a more accurate description of what the authors are referring to?*"

R4. Thank you for pointing out this ambiguity. We have added a footnote to clarify which reads: "In all countries included in the analysis, contributions to social security give workers the right to health insurance. They also include contributions to a pension

to be obtained after retirement, once other eligibility criteria are met.”
C5. "*Discussion (conclusion) The discussion section should be expanded. a. Can the authors generalize about coverage and replacement rates across national income classifications (e.g., upper middle income, high income, etc.)*.

R5. We unfortunately only have 10 countries, so it is hard to generalize what happened across income classifications. We have computed the average coverage and replacement rates across high income, upper middle, and lower middle income countries (Table 1). We only have two high income countries, Chile and Uruguay, which together with Brazil are the countries that imposed the more selective lockdowns. Hence, it is perhaps not surprising that their replacement rate during the lockdown period is the lowest. Perhaps what stands out is a better coverage of pre-existing programs in high income and upper middle income countries, but once again, we only have two lower middle income countries, Bolivia and El Salvador. We believe the country coverage is insufficient to generalize trends across incomes groups and decided to leave it out of the paper.

Table 1: Average percentage of targeted household and replacement rate of COVID-19 Emergency Social Assistance by Country Income classification and Income Quintile

Panel I: Percentage of Targeted Households	A. Preexisting social programs					B. All Transfers				
	Q1	Q2	Q3	Q4	Q5	Q1	Q2	Q3	Q4	Q5
High Income	41.5	25.5	12.5	5.5	2.5	97	74	57.5	39	24.5
Upper Middle Income	46	25	14	8	3	83	65	43	26	12
Lower Middle Income	25	14	12	11	8	95	89	85	79	57
Panel 2: Replacement Rate (including all programs)	C. Median					D. Less than 25%				
	Q1	Q2	Q3	Q4	Q5	Q1	Q2	Q3	Q4	Q5
High Income	30	12	6	5	2	48	81	92	96	94
Upper Middle Income	89	35	22	18	16	9	45	68	75	81
Lower Middle Income	76	28	17	12	8	5	46	76	87	95

Note: Used the World Bank Income classification for the ten countries studied. Panel I (II) shows the simple average of the percentage of targeted households (replacement rate) by income quintiles and High, Upper Middle, and Lower Middle-Income countries. High-income countries include Chile and Uruguay. Upper Middle-Income countries include Peru, Brazil, Ecuador, El Salvador, Dominican Republic, Argentina, and Colombia. Lastly, Bolivia and El Salvador are in the Lower Middle-Income group.

C6. "*b. What can the authors say about budgets for these expansions, including where budgets came from (borrowing, which sectors, etc.), and the percentage of the new programs as compared to overall spending in an average year on social protection?*"

R6. There is surprisingly sparse information on the budget of each specific program (either existing or COVID-19 related programs). Neither we found information of the financing sources of each specific program. What we can report is that all countries experienced a significant increase in their social protection budget during 2020. We added a table (table 2) that shows social expenditures in 2019 and 2020 and write in the text: “There is limited information on the budget allocation to each of these programs

and their sources of financing, but all countries increased significantly their budgets for social protection during the early stages of the pandemic. The raise of budget on social protection between 2019 and 2020 ranges from 8 percent in Uruguay to 225 percent in Dominican Republic (Table 3)”

Table 2: **Government Expenses on social protection by country in 2019 and 2020 (LCU in millions)**

	**2019**	**2020**	**Var %**
Argentina	2,218,102	3,614,937	63.0
Brazil	941,702	1,301,381	38.2
Chile	11,999,419	16,286,275	35.7
Colombia	10,243,156	17,476,454	70.6
Dominican Republic	57,493	187,117	225.5
Ecuador	1137	1558	37
El Salvador	2,569	2,876	12
Peru	6,613	14,046	112.4
Uruguay	19,788	21,445	8.4

Note: The numbers refer to total expenses on "Social Inclusion and Reconciliation" (Colombia), "Social Security" (Argentina), "Social Development" (El Salvador), "Social Benefits" (Dominican Republic and Ecuador) and "Social Protection" (Peru, Chile, Brazil and Uruguay). Sources: Transparency Portal (Colombia, Peru and Uruguay), Fiscal Observatory foundation (Chile), reports by the Ministry of Finance (Dominican Republic and Argentina), National Treasury (Brazil), and Ministry of Economy and Finance (Ecuador, Uruguay and El Salvador). We do not include Bolivia in the table as we did not find updated information on social protection expenses.

C7. "*c. Can the authors suggest some mechanisms to expand coverage to informal workers who “who are not sufficiently poor to benefit from social assistance but lack other automatic stabilizers”*

R7. Thanks for the suggestion. We have expanded the paragraph that now reads as follows “Government responses to the COVID-19 crisis highlight a major structural problem in the region: the fragmented and insufficient coverage of social protection systems. These limitations complicate the delivery of income support to informal workers who are not sufficiently poor to benefit from social assistance but lack other automatic stabilizers, such as unemployment insurance, that could alleviate the impact of temporary shocks. Although the answer to how to protect those who do not contribute to social protection systems is not straightforward, the need to have mechanisms that reach these households during times of negative shocks has become evident. Countries should generalize their information systems to identify the near poor, or vulnerable populations, updating the information on a regular basis. The feasibility and budgetary needs of programs that activate guaranteed minimum income schemes during recessions merits more study in the region. Programs that tackle idiosyncratic income risk will be much more difficult to implement while the levels of informality in the region remain high.”
